# iRGD‐modified exosomes effectively deliver *CPT1A* siRNA to colon cancer cells, reversing oxaliplatin resistance by regulating fatty acid oxidation

**DOI:** 10.1002/1878-0261.13052

**Published:** 2021-07-22

**Authors:** Dan Lin, Haiyang Zhang, Rui Liu, Ting Deng, Tao Ning, Ming Bai, Yuchong Yang, Kegan Zhu, Junyi Wang, Jingjing Duan, Shaohua Ge, Bei Sun, Guoguang Ying, Yi Ba

**Affiliations:** ^1^ Tianjin Medical University Cancer Institute and Hospital National Clinical Research Center for Cancer Tianjin’s Clinical Research Center for Cancer Key Laboratory of Cancer Prevention and Therapy Tianjin China

**Keywords:** chemoresistance, colon cancer, exosomes, FAO, iRGD, oxaliplatin, siRNA

## Abstract

Fatty acid oxidation (FAO) plays a vital role in drug resistance in cancer cells. Carnitine palmitoyltransferase 1A (*CPT1A*), a key enzyme of FAO, is widely recognized as an emerging therapeutic target. Here, we confirmed that *CPT1A* was heterogeneously expressed in colon cancer cells, with a high expression in oxaliplatin‐resistant cells but low expression in oxaliplatin‐sensitive cells, and expression could be increased by oxaliplatin stimulation. In addition, we verified that *CPT1A* was more highly expressed in colon cancer tissues than in noncancerous tissues. Silencing *CPT1A* by siRNA or etomoxir, a specific small‐molecule inhibitor of *CPT1A*, could reverse the sensitivity of drug‐resistant colon cancer cells to oxaliplatin. Subsequently, the combination of oxaliplatin with *CPT1A* inhibition promoted apoptosis and inhibited proliferation. In addition, exosomes were generated with the iRGD peptide on the surface, which showed highly efficient targeting compared with control exosomes *in vivo*. Furthermore, we loaded and therapeutically applied iRGD‐modified exosomes with si*CPT1A* to specifically deliver si*CPT1A* into tumours to suppress FAO. As a consequence, iRGD‐modified exosomes showed the significant inhibition of *CPT1A* in tumour tissues and exhibited the ability to reverse oxaliplatin resistance and inhibit tumour growth by inhibiting FAO with high safety *in vivo*.

## Graphical abstract

Fatty acid oxidation (FAO) plays a vital role in drug resistance in cancer cells. High expression of *Carnitine palmitoyltransferase 1A* (*CPT1A*), a key enzyme in FAO, was observed in colon cancer tissues. Here, we demonstrated that silencing of *CPT1A* by iRGD‐exosome‐si*CPT1A* efficiently suppressed FAO, inhibited tumorigenesis and reversed oxaliplatin resistance in colon cancer. Altogether, iRGD‐exosome‐si*CPT1A* mediated silencing of FAO may serve as an effective approach to treat oxaliplatin‐resistant patient with colon cancer and may propel the clinical application of iRGD‐engineered exosomes for siRNA delivery in cancer treatment.

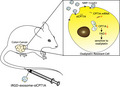

**Abbreviations**
*CPT1A*
: Carnitine palmitoyltransferase 1A
FAO: Fatty acid oxidation
HEK293T/293T: human embryonic kidney epithelial cell line
IHC: immunohistochemical
iRGD/control‐exo: iRGD/control‐exosome
iRGD/control‐exo‐si: iRGD/control‐exo‐si *CPT1A*
NC: negative control
NRP‐1: neuropilin‐1
NTA: nanoparticle tracking analysis
PBS: phosphate‐buffered saline
qRT‐PCR: quantitative reverse transcription‐polymerase chain reaction
siRNAs: small‐interfering RNA
αvβ3: integrin αvβ3

## 1 Introduction

According to 2020 global cancer statistics, colon cancer is the second leading cause of cancer‐related deaths [1, 2]. Chemotherapy is the main strategy in the treatment of advanced colon cancer, and oxaliplatin combined with 5‐fluorouracil (5‐FU) and leucovorin is referred to as FOLFOX, which has been one of the first‐line chemotherapy strategies for colon cancer [3]. However, chemotherapy for colon cancer is compromised as a result of drug resistance. Although response rates have been improved via various combined therapies, such as monoclonal antibodies and chemotherapy, the 5‐year survival rate of metastatic colon cancer is low [4].

According to current research, resistance to oxaliplatin can be caused by multiple mechanisms, including overexpression of chemotherapy resistance‐associated proteins, formation of DNA‐Pt adducts and defects in signalling transduction pathways [5]. Fatty acid oxidation (FAO), is composed of a cyclical series of reactions that result in the shortening of fatty acids and generate NADPH, FADH2 and acetyl‐CoA in each round. Fatty acids provide twice as much ATP as carbohydrates, and in turn, they are preferred nutrients for cancer cells, especially under conditions of nutrient deficiency [6]. As reported, FAO can regulate the proliferation, survival, stemness, drug resistance and metastasis of cancers [7]. Carnitine palmitoyltransferase 1A (*CPT1A*), the key enzyme of FAO, is upregulated in various cancers [8], and it was reported that *CPT1A* has been considered a promising target for cancer therapy [9]. Nevertheless, whether *CPT1A* is correlated with oxaliplatin resistance in colon cancer is unclear.

small‐interfering RNA (siRNA)‐based therapy is in great demands, which can be applied to inhibit specific genes in cancers [10]. However, the clinical application of siRNA is limited by safety and efficiency [11]. Exosomes are potential nanocarriers that protect nucleic acid drugs from damage, and multiple studies have confirmed that engineered exosomes can enhance the tumour‐targeting ability [12]. Various modified proteins have been reported, such as folic acid, platelet‐derived growth factor receptor‐GE1 (PDGF‐GE11) and Lamp2b‐iRGD peptide [13, 14, 15]. iRGD peptide (CRGDKGPDC) helps to promote extravasation and specific penetration by interacting with integrin αvβ3 (αvβ3) or αvβ5 and neuropilin‐1 (NRP‐1) on the tumour vascular endothelium and tumour cells, which shows great potential for tumour targeting [16, 17].

In this study, we first explored the feasibility of delivering siRNA to tumour tissue using iRGD‐engineered exosomes. Second, this study explored the potential correlation between *CPT1A* and oxaliplatin resistance in colon cancer. Finally, we designed iRGD‐engineered exosomes to transport *CPT1A* siRNA specifically to colon cancer cells and tumours, which might efficiently silence *CPT1A* and serve as a potential carrier to reverse oxaliplatin resistance in drug‐resistant colon cancer. In this study, the results indicated that iRGD‐engineered exosomes loaded with si*CPT1A* inhibited FAO, which showed significant tumour targeting and subsequently reversed oxaliplatin resistance in colon cancer *in vivo* and *in vitro*. This might further expand the application of siRNA therapy in clinical practice and provide a novel method for treating oxaliplatin‐resistant colon cancer.

## 2 Materials and methods

### 2.1 Human tissue

Paired colon cancer and noncancerous tissues (13 pairs, a total of 26 samples) were obtained from patients undergoing surgical procedures at the Tianjin Medical University Cancer Institute and Hospital (Tianjin, China). All patient information is listed in Table 1. Both tumour and paracancerous tissues were histologically confirmed by three experienced pathologists. Letters of consent were verified from all patients, and the whole study was approved by the Ethics Committee of Tianjin Medical University Cancer Institute and Hospital, which provided approval numbers for human subjects (Ek2020017). All the study methodologies conformed to the standards set by the Declaration of Helsinki.

**Table 1:** The clinicopathological features of colon cancer patients (*n* = 13).

Characteristics	Number of patients (%) *n* = 13
Age, median (range)	61 (31–76)
Sex	
Female	5 (38.46)
Male	8 (61.54)
Smoking history	
Yes	4 (30.77)
No	9 (69.23)
Family history	
Yes	3 (23.08)
No	10 (76.92)
KPS status	
90–100	9 (69.23)
70–80	4 (30.77)
Tumour location	
Right colon	7 (53.85)
Left colon	6 (46.15)
TNM classification	
I–III	5 (38.46)
IV	8 (61.54)
Tumour grade	
Well differentiation	1 (7.69)
Medium differentiation	8 (61.54)
Low differentiation	4 (30.77)
Metastatic regions	
Liver	3 (23.08)
Liver and Lung	3 (23.08)
Peritoneum	2 (15.38)

### 2.2 Cell culture

The human colon cancer cell lines HCT116 and sw480, the oxaliplatin‐resistant cell lines sw480‐lohp and HCT116‐lohp, and the human embryonic kidney epithelial cell line (HEK293T) were purchased from the Shanghai Institute of Cell Biology of the Chinese Academy of Sciences (Shanghai, China). These four colon cancer cell lines were cultured in RPMI 1640 medium (Gibco, Grand island, NY, USA). HEK293T was cultured in DMEM (Gibco, USA). All of the basal culture media were supplemented with 10% foetal bovine serum (FBS, Gibco, USA) and 1% penicillin/streptomycin. All cells were incubated in a humidified incubator at 37 °C with 5% CO_2_.

### 2.3 Animals

Female nude mice (BALB/c‐nu, 4 weeks) were purchased from the Model Animal Center of Nanjing University and fed in an SPF (specific pathogen‐free) animal facility. All of the experimental procedures were approved by the Institutional Animal Care and Research Advisory Committee of Tianjin Medical University Cancer Institute and Hospital. The ethical approval number for animal is NSFC‐AE‐2020002.

### 2.4 Isolation of exosomes from the medium

Exosomes were isolated from cell culture medium through gradient differential centrifugation. The cell culture was first centrifuged at 300 ***g*** and 3000 ***g*** to remove the cells and then centrifuged at 10,000 ***g*** to remove other vesicles of larger sizes. Finally, the supernatant was centrifuged at 100 000 ***g*** for 80 min using an ultracentrifuge (all steps were performed at 4 °C). Exosomes were resuspended in 50–100 µL of 1X phosphate‐buffered saline (PBS) and stored at 4 °C for 1 week.

### 2.5 PKH26 staining for exosomes

Lipid labelling was performed using PKH26 Red Fluorescent Cell Linker Kits (Sigma, St. Louis, MO, USA). First, exosomes were resuspended in 100 µL of Diluent C. Then, 0.4 µL of PKH26 ethanolic dye solution was added to 100 µL of Diluent C to prepare a dye solution. The exosome suspension (100 µL) was mixed with 100 µL of dye solution by pipetting. After incubating the cell/dye suspension for 4 min, the reaction was stopped by adding 200 µL of serum and incubating for 1 min. Finally, the stained exosomes were washed twice with 1X PBS at 100 000 g for 80 min and resuspended in 1X PBS. Eventually, the stained exosomes were added to the cell culture medium and incubated for 4–8 h before imaging.

### 2.6 Cell transfection

HCT116, HCT116‐lohp, sw480 and sw480‐lohp cells (150,000 cells/well) were seeded into 6‐well plates one day before transfection and transfected according to the manufacturers’ instructions using Lipofectamine 2000 (Invitrogen) and Opti‐MEM (Gibco, USA). For transfection, 100 pmol si*CPT1A* and negative control (NC) were used. Cells were washed twice with 1X PBS first, and then, the transfection reagent was replaced with fresh complete medium 4–6 h after transfection. At 24 h or 48 h after transfection, total RNA or total cell lysate was collected (cells were washed twice with 1X PBS before collection). HEK‐392T cells were cultured in 100 mm dishes and transfected with si*CPT1A* and NC. After 6 h, the culture medium was replaced with DMEM (Gibco, USA) with 10% exosome‐free FBS (Gibco, USA; 48 h) for the isolation of exosomes. All experiments were repeated three times in parallel.

### 2.7 Protein extraction and western blotting

Proteins were collected from cultured cells with SDS lysis buffer (2.2% SDS, 50 mm Tris/HCl pH 6.8, 1 mm PMSF 5.5% glycerol), and the cells were washed twice with 1X PBS before isolation. Proteins from the exosomes were resuspended in SDS lysis buffer after washing in 1X PBS. All protein collection steps were performed at 4 °C. Then, the lysates were heated at 95 °C for 5 min, quantified by a NanoDrop 2000, loaded with β‐Blue (20% β‐mercaptoethanol and 0.08% bromophenol blue) and stored at −80 °C. Thirty to fifty micrograms of protein in each well was used in all experiments (the same quantity of sample in the same experiment was used for each cell line), which was separated via SDS/PAGE and transferred onto polyvinylidene fluoride membranes (PVDF, Roche, Basel, Switzerland). Then, the membranes were incubated with antibodies at 4 °C overnight. The next day, the membranes were incubated with secondary antibodies for 1 h at room temperature. Antibodies, including anti‐CD63 (1:200; Santa Cruz, Dallas, CA, USA; sc‐5275), anti‐TSG101 (1:200; Santa Cruz, sc‐7964), anti‐*CPT1A* (1:1000; Abcam, Cambridge, UK; ab234111), anti‐NRP‐1 (1:1000; Abcam), anti‐Alix (Abcam) and anti‐CD9 (Abcam), were used to analyse the different proteins, and a β‐actin antibody (1:500; Santa Cruz, sc‐47778) was utilized for normalization.

### 2.8 RNA isolation and RT‐qPCR

TRIzol reagent (Invitrogen, Carlsbad, CA, USA) was used to isolate total RNA from cultured cells, exosomes and tissues. A total of 1000 ng of RNA from cultured cells or tissues was used for reverse transcription PCR (Eppendorf AG 22331 Hamburg, Germany). Then, 1 µL of cDNA was added to the real‐time qPCR system. The mRNA levels were normalized to β‐actin. Relative levels of genes were calculated with the 2^−▵▵CT^ method. All genes were assayed at least in triplicate. Primers for *CPT1A* and β‐actin were as follows:

#### 2.8.1 CPT1A primers

*CPT1A*‐ Forward 5′‐TCCAGAGTCCGATTGATTTTTGC‐3′.

#### 2.8.2 β‐actin primers

actin‐Forward 5′‐GGCTGTGCTATCCCTGTACG‐3′.

actin‐Reverse 5′‐CTTGATCTTCATTGTGCTGGGTG‐3′.

### 2.9 Transmission electron microscopy (TEM)

Exosomes were suspended in 1X PBS, and then, a drop of the exosomal suspension was added to the copper net, dried in the dark for 2 min, after which the edge liquid was absorbed. Next, a drop of staining agent (2% phosphoric acid, pH 6.5) was added, followed by incubation for 2 min and drying for 9 min with an incandescent lamp. Finally, the exosomes were observed via an FEI Tecnai T20 transmission electron microscope (Thermo, Waltham, MA, USA) (120 kV).

### 2.10 Nanoparticle tracking analysis

The sizes and numbers of exosomes were detected via the NanoSight NS300 system (NanoSight Technology, Malvern, UK). Exosomes were resuspended in 1X PBS and further diluted 1000‐fold to achieve between 20 and 100 objects per frame. Then, every sample was injected into sample chambers, which were visualized with a 488 nm laser and a high‐sensitivity sCMOS camera. All the data were analysed by the Nanoparticle tracking analysis (nta) analytical software.

### 2.11 IHC staining assay

Paraffin‐embedded tissue samples of paired colon cancer and adjacent tissues were sectioned and stained with a 1 : 1000 dilution of anti‐*CPT1A* antibody (ab234111), 1 : 500 dilution of anti‐NRP‐1 antibody (Abcam), 1 : 100 dilution of anti‐αvβ3 antibody (Bioss, bs‐1310R) and 1 : 500 dilution of anti‐Ki‐67 antibody (ab92742). A DAB system (Zhongshan Jinqiao, China) was used to identify positive staining. Five regions were selected randomly for each specimen.

### 2.12 ATP measurement

Cells were seeded in 96‐well plates, and control wells were also prepared to obtain background luminescence. A CellTiter‐Glo Luminescent Cell Viability Assay (Promega, Madison, WI, USA) was used for ATP measurement. The plate and its contents were stored at RT for approximately 30 min. Then, 100 µL of CellTiter‐Glo reagent was added to each well, followed by mixing for 2 min on a shaker to induce cell lysis and incubation at RT for 10 min to stabilize the luminescent signal. Finally, the luminescence was recorded via a microplate reader (BioTek Synergy H1, Winooski, VT, USA).

### 2.13 CCK‐8 cell viability assay

A CCK‐8 kit (My Biotech, Ueberherrn, Germany) was used for the cell viability test. All cells were seeded into 96‐well plates with different pretreatments. Then, different concentrations of oxaliplatin (0 µg·mL^−1^–80 µg·mL^−1^) were added. After incubation for 48 h, 10 μL of CCK‐8 reagent was added and incubated at 37 °C for 2–3 h. A microplate reader (Thermo) was used to detect optical density (OD) values at a wavelength of 460 nm. The assay was performed at least in triplicate. Cell viability was calculated by the following formula:

$$\text{Inhibition}\;\text{rates}\;=\;\left({\text{OD}}_{\text{control}}\;‐\;{\text{OD}}_{\text{experiment}}\right)\;/\;\left({\text{OD}}_{\text{control}}\;‐\;{\text{OD}}_{\text{blank}}\right)\;*\;100\%$$

### 2.14 EdU cell proliferation assay

The proliferation ability was determined by the EdU proliferation assay (RiboBio). EdU is a thymidine analogue, and its attached alkyne group is rare in natural compounds. It can be incorporated as thymine (T) into the DNA molecule being synthesized during DNA replication. Based on the specific reaction of Apollo® fluorescent dyes with EdU, DNA replication activity can be directly and accurately detected. After different treatments of HCT116‐lohp and sw480‐lohp cells (etomoxir 150 μm, oxaliplatin 2 μg·mL^−1^. siRNA/NC or combined according to the experiment), all steps were carried out in accordance with the instructions of the EdU proliferation assay (RiboBio). EdU (50 μm) was added to the cells, which were then incubated for 5 h, fixed, filtered and stained according to the instructions. Finally, the results were imaged by a Leica microscope (blue represents all cells, red represents proliferating cells).

### 2.15 Flow cytometry

HCT116‐lohp and sw480‐lohp cells were pretreated differently (etomoxir 150 μm, oxaliplatin 2 μg·mL^−1^, siRNA/NC or combined according to the experiment) for 48 h. An annexin V‐FITC/PI staining kit (BD Biosciences) was used for this test. First, cells were washed with 1XPBS twice and resuspended in 1X binding buffer. Then, the cells were stained with annexin V‐FITC for 10 min and PI for 5 min in the dark. Finally, apoptotic cells were quantified using flow cytometry (BD Biosciences, Franklin Lake, NJ, USA).

### 2.16 BODIPY‐493/503 staining assay

Cell lines with different treatments were pretreated with oleic acid (OA, 200 μm) for 24 h and then cultured in low glucose media RPMI 1640 for additional 48 h. Then, cells were stained with BODIPY‐493/503 (dyes of fatty acids, 1 µg·mL^−1^) for 30 min and DAPI (1 : 1000 diluted by 1 X PBS) for 10 min. Finally, all groups were imaged using confocal microscopy (Zeiss, Jena, Germany).

### 2.17 Establishment of tumours in nude mice

HCT116‐lohp cells were cultured in advance to an appropriate density. A total of 2 × 10^7^ HCT116‐lohp cells were subcutaneously injected into the left inguinal fold of the mice. In addition, the tumours were sized every 2 days. Different exosome treatments began when the tumours grew to the proper size (the length and width of tumours were about 8 mm). Subsequently, the tumours were removed after a month and divided into small pieces 2 mm in diameter. Volume of tumours was calculated by the following formula: Volume = Length × Width × Width/2.

### 2.18 Statistical analyses

All experiments were repeated at least three times in parallel, and the data are expressed as the mean ± SEM. A *P* value < 0.05 was considered statistically significant by using the Student’s t‐test: *P < 0.05, **P < 0.01, ***P < 0.001 and ****P < 0.0001.

## 3 Results

### 3.1 Establishment and characterization of iRGD‐modified exosomes and identification of colon cancer as a potential target

**Fig. 1:**
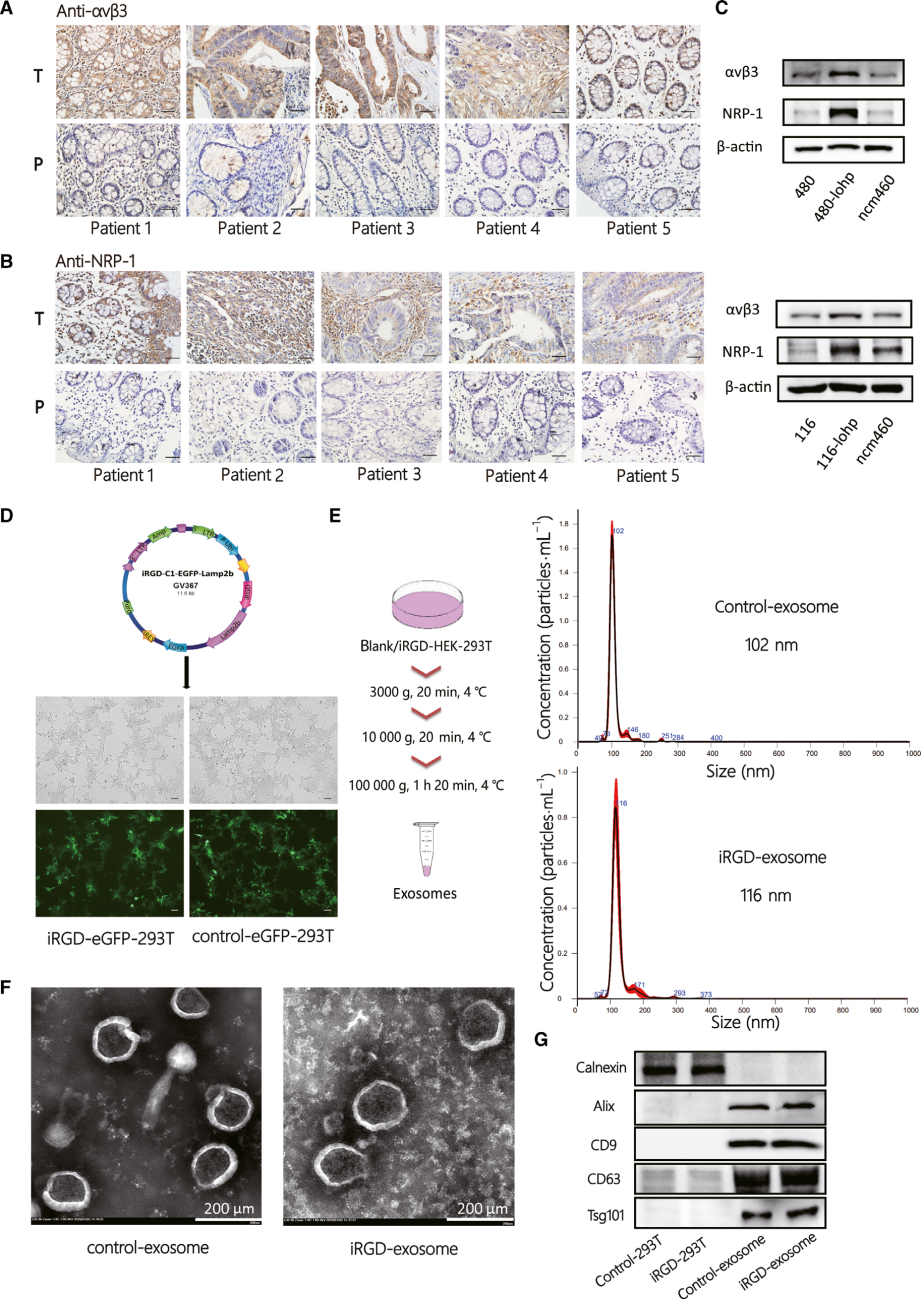
Establishment and characterization of iRGD‐modified exosomes and identification of colon cancer as potential target. (A–B) IHC staining of the paraffin‐embedded human colon cancer tissues and paired normal tissues to identify the expression of αvβ3 (A) and NRP‐1 (B) [total *n* = 13, 5 of 13 are shown in (A, B)], and the others are shown in Fig. S1A‐B). (C) Western blotting (WB) analysis of αvβ3 and NRP‐1 expression in colon cancer cell lines and normal intestinal epithelial cell lines. (D) The vital composition of the plasmid of IRGD‐C1‐EGFP‐Lamp2b and Iimage of iRGD/control‐eGFP‐293T cell lines under a fluorescence microscope (scale bar = 100 μm). (E) Schematic diagram of exosome isolation steps and NTA validation of iRGD/control exosome (iRGD/control‐exo) from the cell supernatant. (F) Transmission electron microscopy (TEM) confirmation of iRGD/control‐exo from the cell supernatant (scale bar = 200 nm). (G) Western blotting of exosome markers, including calnexin, Alix, CD9, CD63 and Tsg101.

According to the targeting theory of the iRGD peptide, the peptide can realize extravasation and tumour‐specific penetration by binding αvβ3 or αvβ5 and NRP‐1 on the tumour vascular endothelium and various tumour cells. To identify whether colon cancer was the preferred target, we chose 13 paired cancerous and noncancerous tissues to assess the expression of αvβ3 and NRP‐1. As expected, a high level of expression of αvβ3 and NRP‐1 was clearly observed in cancer tissues compared with noncancerous tissues (Figs 1A,B and S1A,B).

The sw480‐lohp/HCT116‐lohp cells, oxaliplatin‐resistant colon cancer cell lines, showed a higher level of expression of αvβ3 and NRP‐1 than the normal colon cell line ncm460 (Fig. 1C). Thus, we identified colon cancer as a potential target for the iRGD peptide. Next, to successfully generate iRGD exosome, we infected HEK293T cells with virus for loading iRGD‐C1‐eGFP‐Lamp2b or the negative control (control‐C1‐eGFP‐Lamp2b) and confirmed iRGD/control‐eGFP‐293T under a fluorescence microscope (Fig. 1D). Then, control exosome (control‐exo) and iRGD exosome (iRGD‐exo) were isolated from the cell culture medium through gradient differential centrifugation. The sizes were measured by NTA, which showed that control exosome or iRGD exosome had similar sizes at approximately 102–116 nm (Fig. 1E). Under transmission electron microscopy (TEM), control exosome or iRGD exosome were discovered to have similar shapes (Fig. 1F). Moreover, we characterized representative markers of exosomes, such as Alix, CD9, CD63, Tsg101 and negative marker of exosomes, calnexin (Fig. 1G). These results indicated that modification with the iRGD peptide did not change the original traits of the exosomes and that colon cancer could be a potential target of iRGD exosome.

### 3.2 Enhanced cellular uptake of iRGD‐exosome in colon cancer compared with control exosome *in vitro* and *in vivo*

**Fig. 2:**
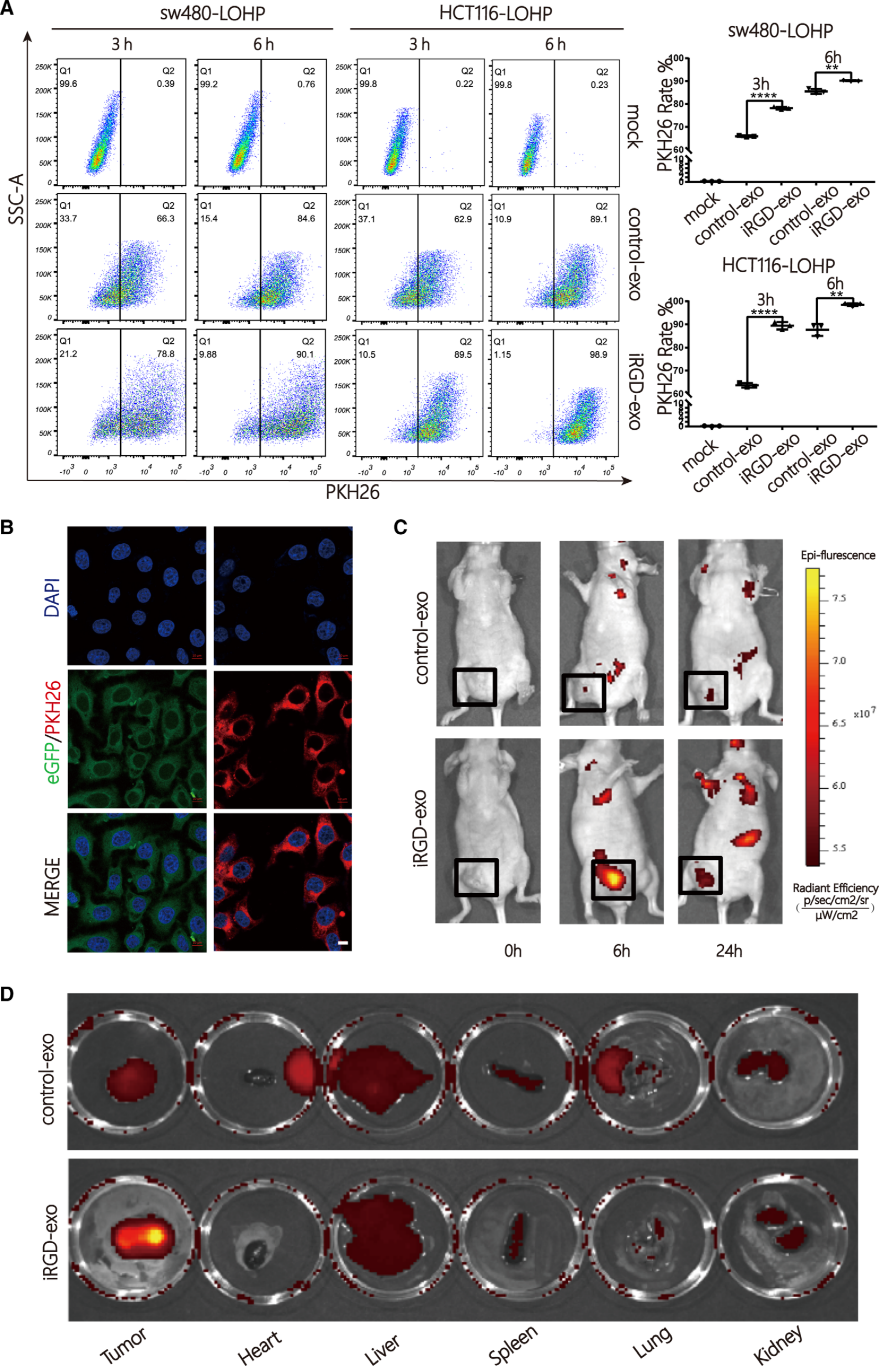
Enhanced cellular uptake of iRGD‐exosomes in colon cancer compared with control‐exosomes in vitro and in vivo. (A) Analysis rate of sw480‐lohp/HCT116‐lohp cells containing PKH26 after treatment with PKH26‐labelled iRGD/control‐exo by flow cytometry (*n* = 3, mean ± SEM, *t*‐test, **P < 0.01, and ****P < 0.0001). (B) Exosomes stained with PKH26 could be fused into colon cancer cells (scale bar = 10 μm). (C) Mice bearing HCT116‐lohp tumours were given intravenous injection of DiR‐labelled iRGD/control‐exos (200 μg per mouse). In vivo fluorescence signals were recorded for 24 h postinjection by IVIS SPECTRUM. Fluorescence was detected at the tumour sites (black boxes). (D) Fluorescence imaging of major isolated organs, including the tumour, heart, liver, spleen, lung and kidney, from tumour‐bearing mice at 6 h after intravenous injection with DiR‐labelled iRGD/control‐exo (200 μg per mouse).

To distinguish the different affinities of exosomes for colon cancer, we loaded control exosome or iRGD‐exosome with the PKH26 dye first, and cocultured them with sw480‐lohp and HCT116‐lohp cells for 3 h and 6 h (Fig. 2A). According to the results, iRGD‐exosome had a higher affinity than the control‐exosome for both sw480‐lohp and HCT116‐lohp cells, confirming that the iRGD peptide dramatically enhanced the binding capability of exosomes to target cells and that the recipient cells received more exosomes over time. Exosomes with eGFP or labelled with PKH26 were successfully phagocytized into recipient cells as determined by a laser scanning confocal microscope (Fig. 2B). In vivo, we prepared HCT116‐lohp tumour‐bearing nude mouse models. To visualize the distribution of exosomes in vivo, control‐exosome or iRGD‐exosome was labelled with DiR dye and injected intravenously through the tails. The locations of exosomes were monitored at 0 h, 6 h and 24 h using an IVIS SPECTRUM in vivo optical imaging system. According to the results, the iRGD‐exo was able to target the tumours at approximately 6 h after injection (Fig. 2C). To further analyse the distribution of the injected iRGD‐exo, we removed the organs of interest 6 h after injection, and fluorescence imaging showed that the iRGD‐exo had strong fluorescence signals in the tumour tissue while the control‐exos did not (Fig. 2D). These data suggested a more efficient uptake of iRGD‐exo in vitro and in vivo compared with control‐exo.

### 3.3 *CPT1A* might be a promising target to reverse oxaliplatin resistance in colon cancer

**Fig. 3:**
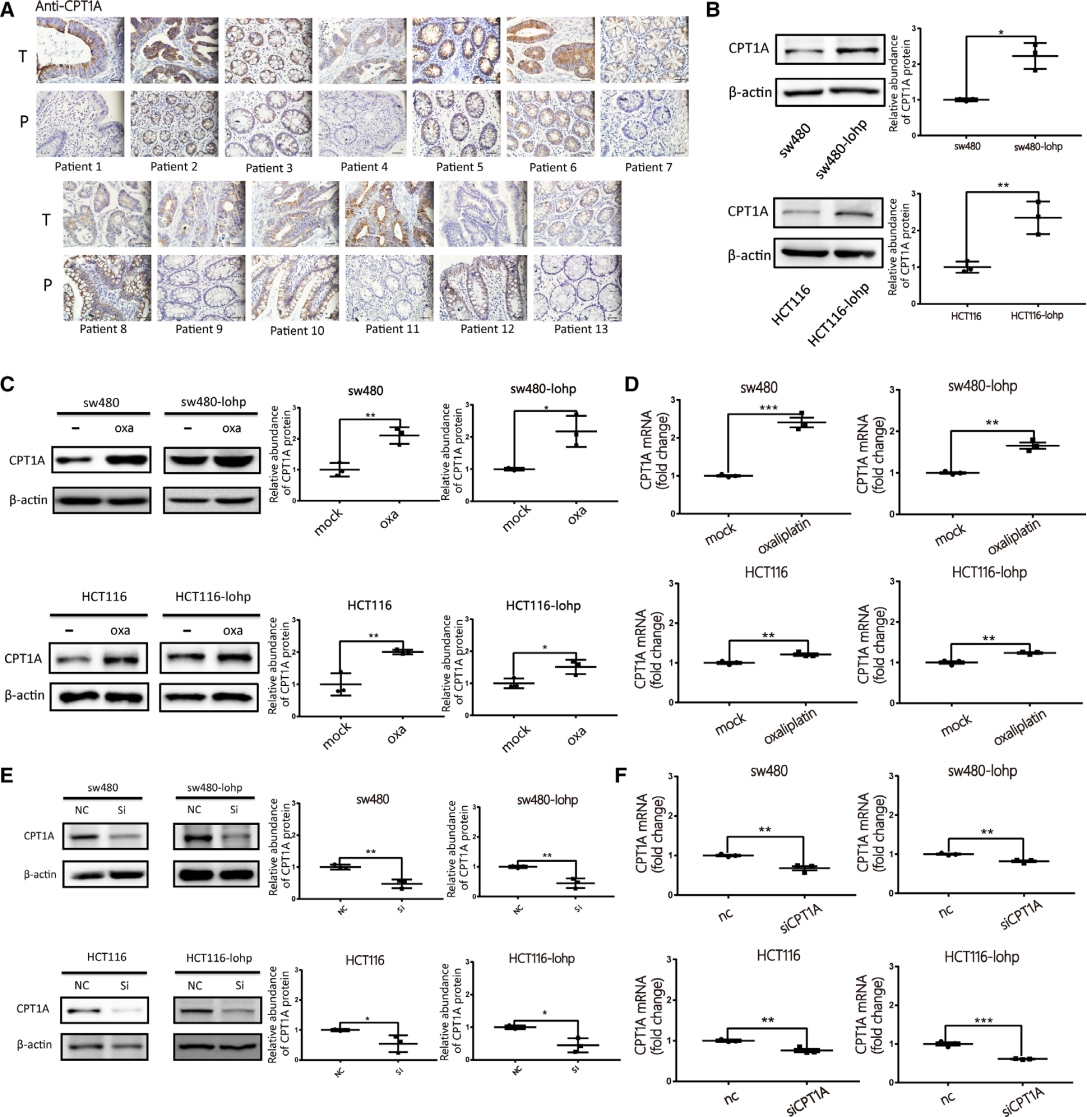
*CPT1A* might be a promising target to reverse oxaliplatin resistance in colon cancer. (A) Differential expression of *CPT1A* was identified by IHC staining in human colon cancer tissues and paired normal tissues (*n* = 13). (B) Western blotting analysis of *CPT1A* expression in different colon cancer cell lines and grey value analysis of *CPT1A* protein (*n* = 3, mean ± SEM, *t*‐test). (C–D) Expression of *CPT1A* after oxaliplatin stimulation by western blotting (C) and qRT‐PCR (D); all *Y*‐axes indicate the fold change of *CPT1A* (*n* = 3, mean ± SEM, *t*‐test). (E–F)Verification of *CPT1A* expression after siRNA transfection by western blotting (E) and qRT‐PCR (F), all y‐axes indicate the fold change of *CPT1A* (*n* = 3, mean ± SEM, *t*‐test). *P < 0.05; **P < 0.01; ***P < 0.001.

*CPT1A*, representing the key enzyme of FAO, has emerged as a promising target for cancer therapy. The high expression of *CPT1A* is related to apoptosis, proliferation and drug resistance among various cancers. However, no research has identified whether *CPT1A* is related to oxaliplatin resistance in colon cancer. We first confirmed the expression of *CPT1A* in 13 pairs of colon cancer tissues, and it showed higher levels in cancer tissue (Fig. 3A). The resistance to oxaliplatin in colon cancer cell lines was identified in Fig. S1C. Oxaliplatin‐resistant cell lines, including sw480‐lohp and HCT116‐lohp, expressed higher *CPT1A* (Fig. 3B) and oxaliplatin (2 µg·mL^−1^), stimulated the protein and mRNA levels of *CPT1A* in these cell lines (Fig. 3C,D). Thus, *CPT1A* could be a target related to oxaliplatin resistance. To knock down *CPT1A*, siRNA was designed to block the expression of *CPT1A* in colon cancer cell lines (Fig. 3E,F). siRNA was able to silence *CPT1A*, which might be a way to reverse oxaliplatin resistance.

### 3.4 Inhibition of *CPT1A* suppressed FAO and reversed oxaliplatin resistance in colon cancer

**Fig. 4:**
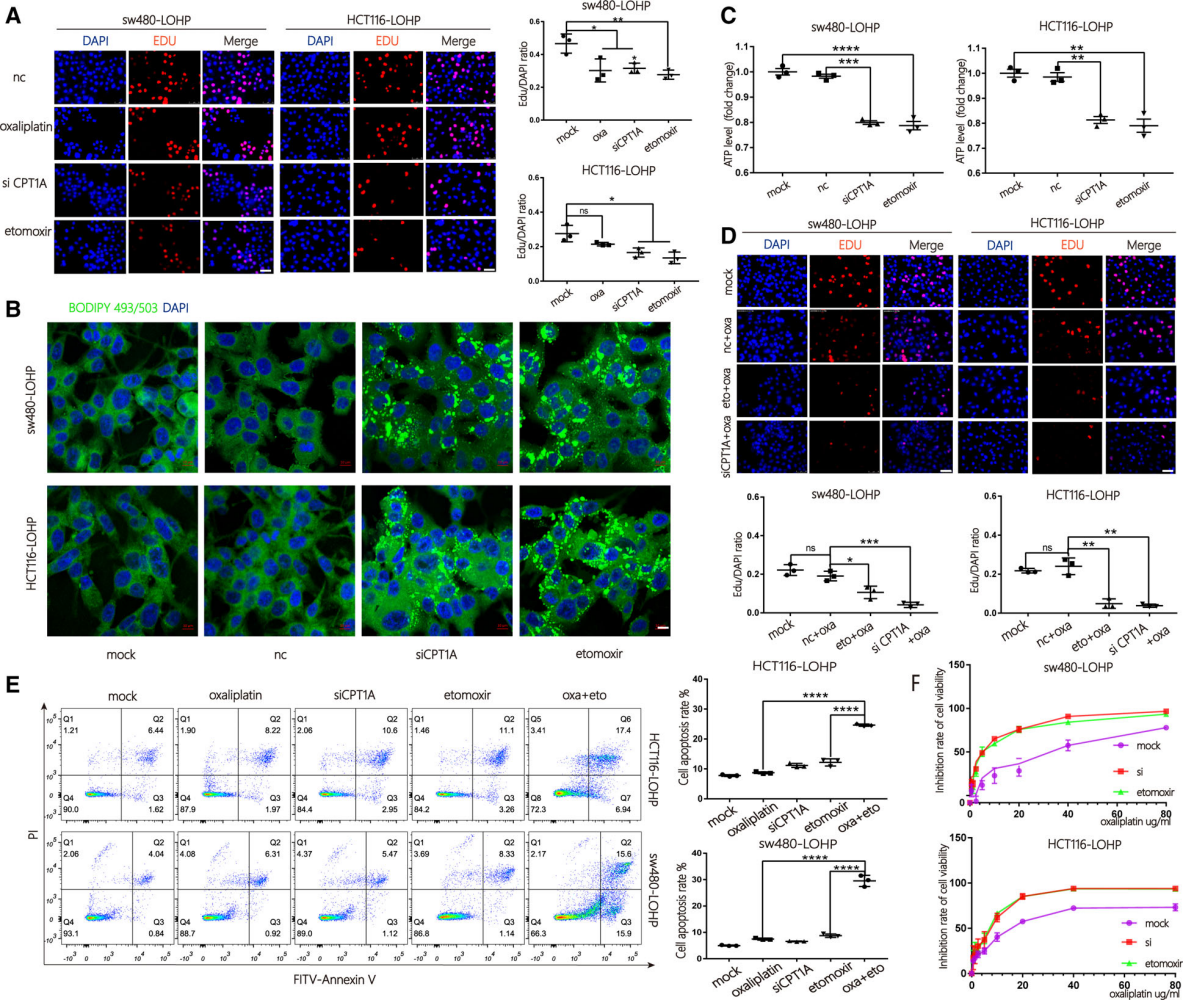
Inhibition of *CPT1A* suppressed FAO and reversed oxaliplatin resistance in colon cancer. (A) An EdU assay was used to evaluate the proliferation of sw480‐lohp/HCT116‐lohp cells after different treatments; red indicates newly proliferating cells (scale bar = 50 μm, *n* = 3, mean ± SEM, *t*‐test). (B) Cell lines with *CPT1A* inhibited by siRNA or etomoxir were pretreated with oleic acid (OA, 200 μm) for 24 h and then cultured in low glucose media RPMI 1640 for an additional 48 h. Cells were stained with BODIPY‐493/503 (dyes of fatty acids, 1 µg·mL^−1^, green) and DAPI (blue) and imaged by confocal microscopy (scale bar = 10 μm). (C) Relative ATP production of cells treated with si*CPT1A* or etomoxir was analysed by a CellTiter‐Glo Luminescent Cell Viability Assay (*n* = 3, mean ± SEM, *t*‐test). (D) Proliferation analysis of sw480‐lohp/HCT116‐lohp cells treated with a combination of oxaliplatin and *CPT1A* inhibition by EdU assay (scale bar = 50 μm, *n* = 3, mean ± SEM, t‐test). (E) Flow cytometry analysis of apoptosis in sw480‐lohp/HCT116‐lohp cells treated with si*CPT1A*, oxaliplatin, etomoxir or etomoxir combined with oxaliplatin (*n* = 3, mean ± SEM, *t*‐test). (F) CCK‐8 test of the inhibition ratio by oxaliplatin in sw480‐lohp/HCT116‐lohp cells treated with si*CPT1A* or etomoxir. *P < 0.05; **P < 0.01; ***P < 0.001; ****P < 0.0001.

To assess the function of *CPT1A*, an EdU assay was performed to confirm the ratio of newly proliferating colon cancer cells after blocking *CPT1A* (Fig. 4A). Etomoxir is a small‐molecule inhibitor of *CPT1A* that acts as a positive control. Based on the results, both si*CPT1A* and etomoxir suppressed proliferation of sw480‐lohp and HCT116‐lohp.

To continue exploring the mechanisms, we first treated sw480‐lohp and HCT116‐lohp cells with OA (oleic acids) for 24 h and then cultured them in low glucose media for an additional 48 h to allow sufficient utilization of fatty acids. After BODIPY‐493/503 staining, green signals were observed within the cytoplasm, which suggests that si*CPT1A* or etomoxir inhibited the utilization of OA in sw480‐lohp and HCT116‐lohp cells (Fig. 4B). Inhibition of *CPT1A* also decreased total ATP levels in cells (Fig. 4C). In addition, the combination of siRNA/etomoxir with oxaliplatin achieved a higher proliferation suppression than oxaliplatin alone (Fig. 4D). Moreover, inhibition of *CPT1A* helped oxaliplatin to increase the apoptosis of cells, as shown by flow cytometry (Fig. 4E). Afterwards, we pretreated sw480‐lohp and HCT116‐lohp cells with si*CPT1A* or etomoxir for 24 h and then added gradient concentrations of oxaliplatin to each well of the two cell lines for 48 h. We tested cell viability via the CCK‐8 reagent and calculated the inhibition rate. As expected, inhibition of *CPT1A* increased the sensitivity of sw480‐lohp and HCT116‐lohp cells to oxaliplatin (Fig. 4F). These results confirmed that suppression of *CPT1A* by siRNA or etomoxir could both block the utilization of fatty acids and the production of ATP, which might result in the regulation of apoptosis and proliferation and eventually reverse oxaliplatin resistance in colon cancer.

### 3.5 Improved efficacy of si*CPT1A* delivered by iRGD‐exo in colon cancer

**Fig. 5:**
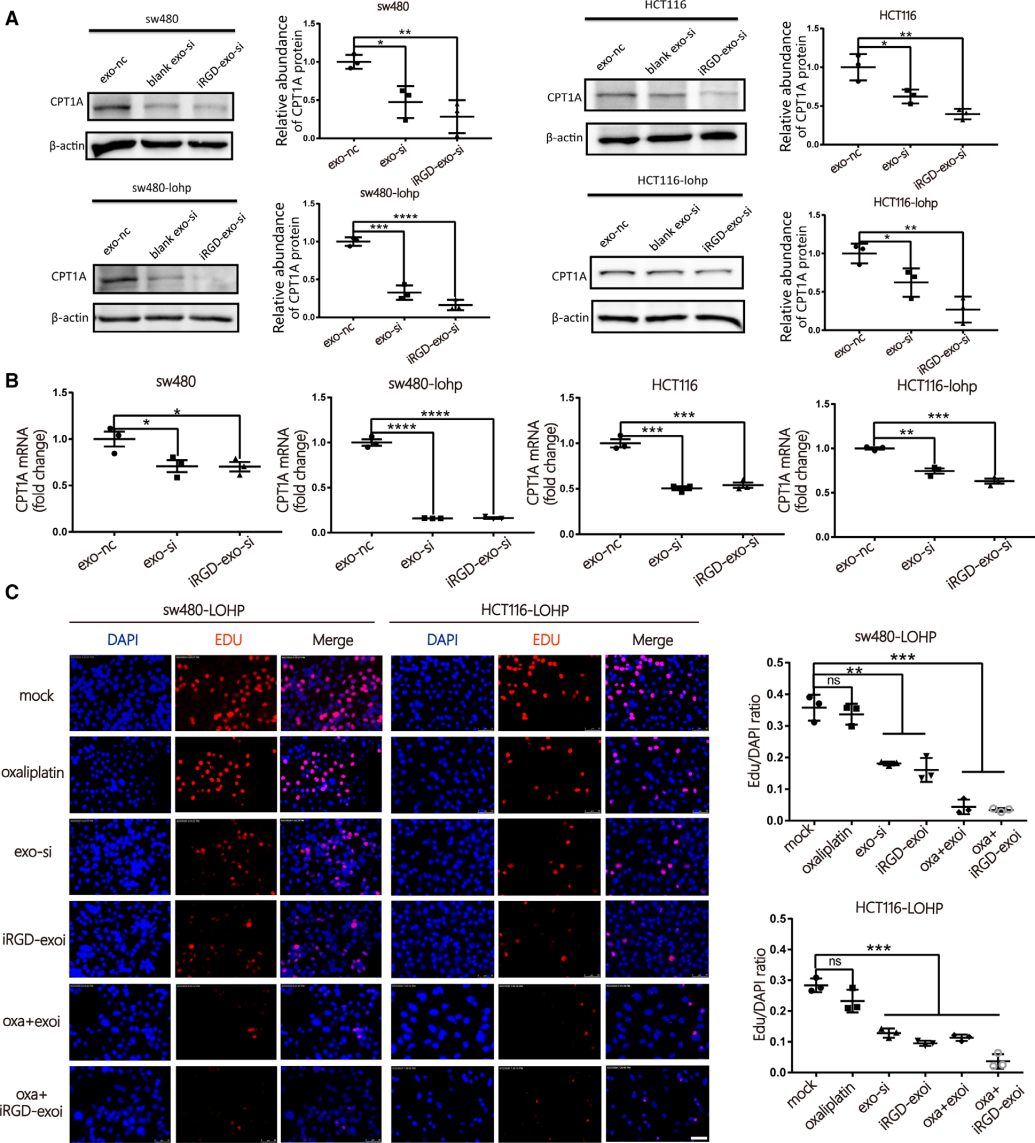
Improved efficacy of si*CPT1A* delivered by iRGD‐exos in colon cancer. (A–B) Expression of *CPT1A* in colon cancer cell lines after coculture with iRGD/control‐exo‐si by western blotting (A) and qRT‐PCR (B) (*n* = 3, mean ± SEM, *t*‐test). (C)Evaluation of the rate of proliferating cells after coculture with iRGD/control‐exo‐si or oxaliplatin by EdU assay (scale bar = 50 μm, *n* = 3, mean ± SEM, *t*‐test). *P < 0.05; **P < 0.01; ***P < 0.001; ****P < 0.0001.

**Fig. 6:**
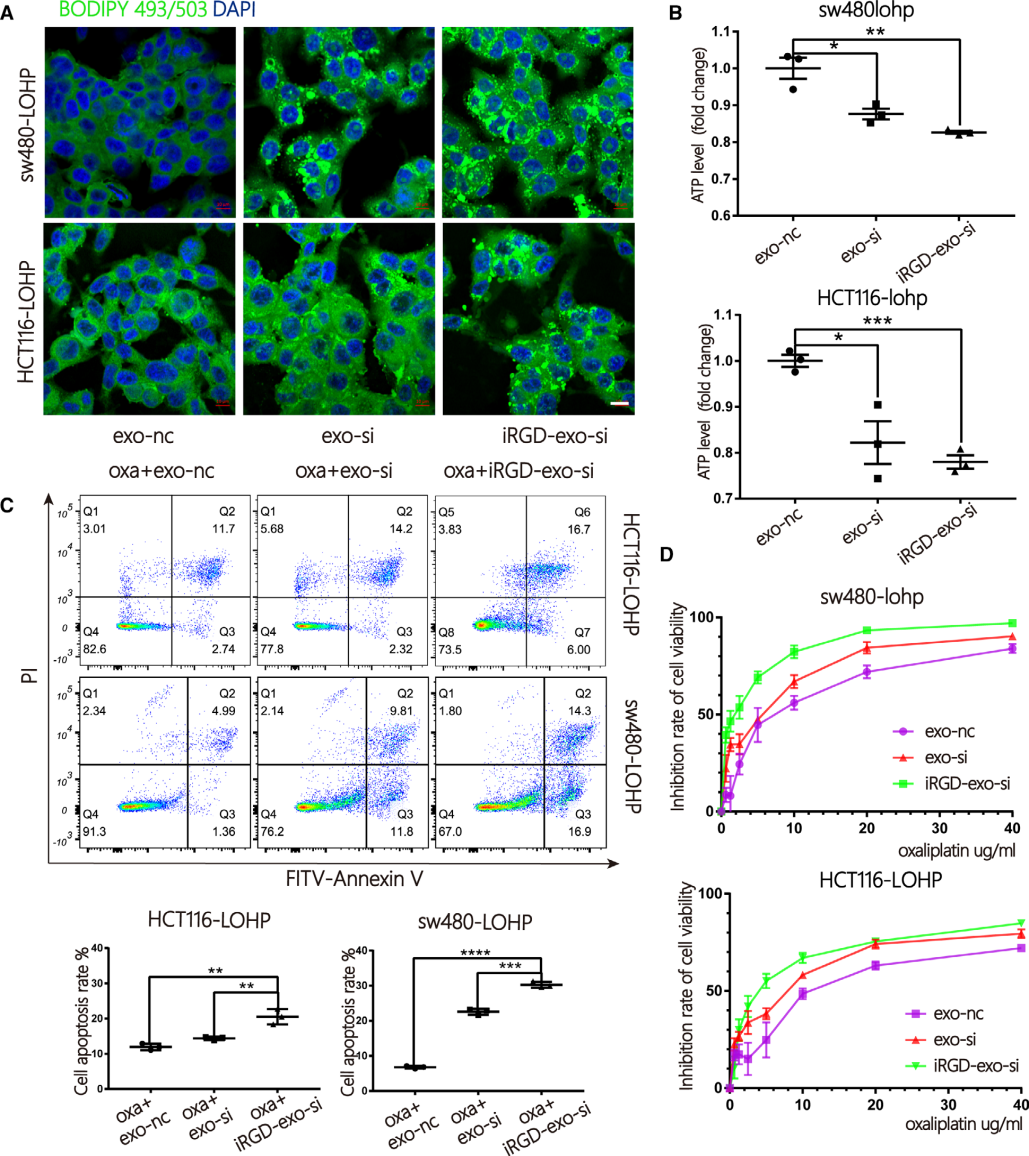
Improved inhibition of FAO by iRGD‐exo‐si in colon cancer. (A) Cells treated with iRGD/control‐exo‐si were stained with BODIPY‐493/503 (dyes of fatty acids, 1 µg·mL^−1^, green) and DAPI (blue) and imaged by confocal microscopy (scale bar = 10 μm). (B) A CellTiter‐Glo Luminescent Cell Viability Assay was used to test the total ATP production of cells treated with iRGD/control‐exo‐si (*n* = 3, mean ± SEM, *t*‐test). (C) Apoptosis ratio by flow cytometry in sw480‐lohp/HCT116‐lohp cells treated with iRGD/control‐exo‐si and oxaliplatin (*n* = 3, mean ± SEM, *t*‐test). (D) CCK‐8 assay of the inhibition ratio by oxaliplatin in sw480‐lohp/HCT116‐lohp cells treated with iRGD/control‐exo‐si (*n* = 3, mean ± SEM, *t*‐test). *P < 0.05; **P < 0.01; ***P < 0.001; ****P < 0.0001.

Due to the limitations of siRNA application in the clinic, exosomes are recognized to be promising delivery vehicles of siRNA. To load siRNA into exosomes, iRGD/control‐293T cells were transfected with si*CPT1A* and si‐NC and incubated with exosome‐free DMEM for 48 h. The iRGD/control‐exosome‐si*CPT1A* (iRGD/control‐exo‐si) were collected from cultures of iRGD/control‐293T cells by centrifugation. Next, exosomes were cocultured with colon cancer cells, and it was confirmed by quantitative reverse transcription‐polymerase chain reaction (qRT‐PCR) and western blot that iRGD‐exo‐si significantly downregulated the expression of *CPT1A* more efficiently than control‐exo‐si (exo‐si) (Fig. 5A,B). Consistent with these results, iRGD‐exo‐si in combination with oxaliplatin inhibited proliferation more efficiently than control‐exo‐si in colon cancer cells (Fig. 5C). In addition, iRGD‐exo‐si more strongly inhibited the utilization of fatty acids and production of ATP in cells (Fig. 6A,B). Similarly, iRGD‐exo‐si showed stronger potential to inhibit *CPT1A* to help oxaliplatin promote apoptosis (Fig. 6C). Finally, through the CCK‐8 test, iRGD‐exo‐si demonstrated a better effect on the reversal of oxaliplatin resistance in colon cancer (Fig. 6D). In conclusion, iRGD‐exo‐si showed enhanced efficiency in knocking down *CPT1A* compared with control‐exo‐si, which might provide a new method for delivery of siRNA targeting for colon cancer.

### 3.6 iRGD‐exo loaded with si*CPT1A* showed better therapeutic efficacy than control‐exo in oxaliplatin‐resistant colon cancer *in vivo*

**Fig. 7:**
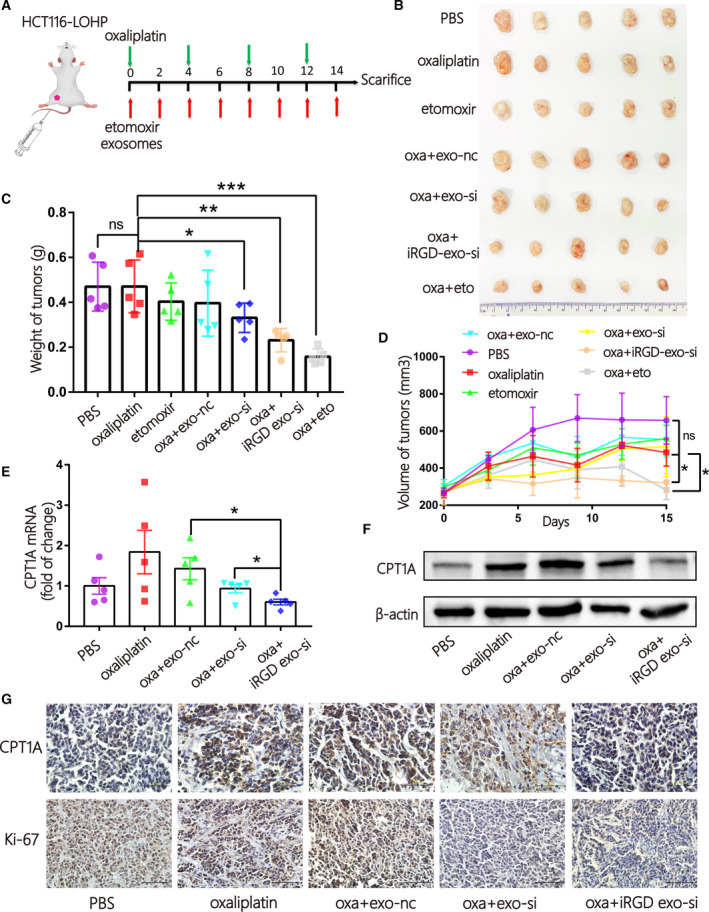
iRGD‐exo loaded with si*CPT1A* showed better therapeutic efficacy than control‐exo in oxaliplatin‐resistant colon cancer in vivo. (A) A flow diagram of the vivo experimental design. (B) Picture of xenografted tumours from mice after sacrifice (*n* = 35, 5/per group). (C‐D) Quantitative analysis the weight (C) and volume (D) of tumours (*n* = 5, mean ± SEM, *t*‐test). (E‐F) qRT‐PCR (E) and WB (F) quantification of *CPT1A* tumours in tumours in the different groups. (*n* = 5, mean ± SEM, *t*‐test). (G) The expression of *CPT1A* and Ki‐67 in tissues obtained from the different groups was identified by IHC (scale bar = 1 mm, *n* = 5). *P < 0.05; **P < 0.01; ***P < 0.001.

Based on these in vitro results, we established an oxaliplatin‐resistant colon cancer model by injecting HCT116‐lohp cells subcutaneously (*n* = 35, divided into 7 groups). Treatment began when the tumours grew to the proper size, and different treatments are shown in Fig. 7A. One hundred micrograms of different exosomes (resuspended in 100 μL PBS) or PBS was injected through the tail veins every 2 days, and intraperitoneal injections of 8 mg·kg^−1^ oxaliplatin every 4 days or 15 mg·kg^−1^ etomoxir every 2 days were performed. As shown in Fig. 7B, the combination of oxaliplatin and etomoxir had the best efficacy in inhibiting tumour growth. However, etomoxir exhibited toxicity as reported. The combination of oxaliplatin with iRGD‐exo‐si had a better curative effect than exo‐si without a strong toxicity (Fig. 7C,D). The inhibition rate of *CPT1A* is shown in Fig. 7E,F. Oxaliplatin stimulated the expression of *CPT1A*, and iRGD‐exo‐si showed benefits in blocking *CPT1A* compared with exo‐si. Immunohistochemical (IHC) was used to identify the expression of *CPT1A* and Ki‐67, and the proliferation was suppressed in the combination of oxaliplatin and iRGD‐exo‐si treatment in Fig. 7G. In conclusion, iRGD‐exo‐si combined with oxaliplatin could be a preferred method to reverse drug resistance compared with the application of siRNA only in colon cancer.

## 4 Discussion

Tumour targeting is a key performance criterion that nanocarriers are supposed to possess. Exosomes are excellent natural carriers because of their low toxicity and low immunogenicity. The iRGD peptide not only strictly binds to αv integrins that are highly expressed on tumour cells but also facilitates vascular or tissue penetration of drugs [16]. As reported, exosomes engineered by the iRGD peptide have shown great affinity for breast cancer [14]. In this study, we successfully established and identified iRGD‐modified exosomes (iRGD‐exosome), and it was confirmed that both αvβ3 and NRP‐1 were highly expressed in colon cancer and cell lines, which demonstrated that colon cancer could be a potential target for iRGD‐exosome. As expected, iRGD‐exosome showed enhanced cellular uptake compared with control‐exosome in vitro, and iRGD exosomes focussed on the tumour site distinctly within 24 h in vivo after intravenous injection. In this manner, iRGD exosome enabled significant targeting to colon cancer, which could be targeting nanocarrier of siRNA for further application in clinic.

Chemotherapy is the main strategy for patients with advanced colon cancer. More evidence has confirmed that *CPT1A* might be a promising target to reverse sensitivity to chemotherapy in cancers [18, 19]. It was reported that mesenchymal stem cells might promote chemoresistance of gastric cancer to FOLFOX by upregulating FAO [20]. In this study, *CPT1A* was found to be highly expressed in colon cancer tissues compared with adjacent tissues. *CPT1A* was highly expressed in oxaliplatin‐resistant cells but not in oxaliplatin‐sensitive cells. More importantly, the results showed that the expression of *CPT1A* increased significantly after oxaliplatin stimulation, which implied that *CPT1A* might be a vital target to reverse oxaliplatin resistance in colon cancer. To further confirm this hypothesis, we checked the IC50 of cells directly after silencing *CPT1A* with siRNA or etomoxir and it showed that blocking *CPT1A* reversed oxaliplatin resistance in colon cancer, which could be a novel method for reversal of oxaliplatin resistance. However, CPT1 inhibitors, such as etomoxir and perhexiline, exhibit hepatotoxicity, which limits the development of clinical applications [21].

To develop RNAi‐based therapeutics, it is urgent to create nanocarriers with low toxicity but high tumour permeation [22]. Several studies have exploited the potential application of exosomes after being engineered for drug delivery [23]. Exosomes have been employed to deliver RNA drugs to specific locations in vivo, especially tumour tissues, and have shown great potential as naturally occurring nanocarriers [24, 25]. Thus, our experiment used iRGD‐exosome to carry *CPT1A* siRNA to suppress FAO and reverse oxaliplatin resistance in colon cancer. According to the results, oxaliplatin combined with inhibition of *CPT1A* suppressed proliferation and facilitated apoptosis in oxaliplatin‐resistant cells, possibly because it could obstruct the metabolism of fatty acids and reduce ATP production. iRGD‐exo‐si showed greater suppression of *CPT1A* than exo‐si *in vitro* and *in vivo*, which suggested that iRGD exosome was a superior candidate for specific siRNA delivery to colon cancer. In vivo, iRGD‐exo‐si sensitized oxaliplatin therapy in an oxaliplatin‐resistant model, with low toxicity and high tumour targeting, and it might solve the difficulty that RNAi‐based therapeutics have met and propel the application of modified exosomes in cancer therapy.

## 5 Conclusions

**Fig. 8:**
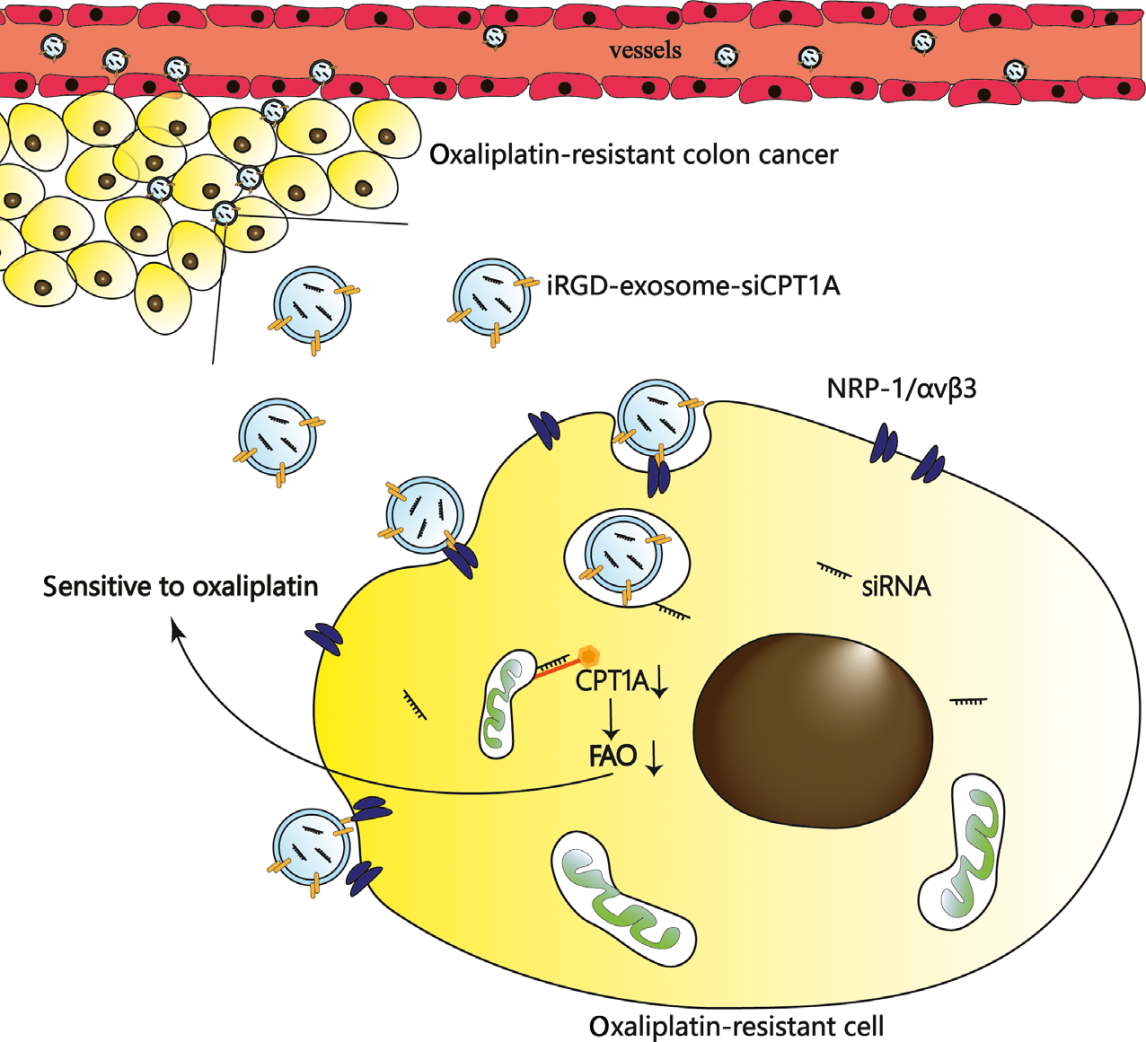
Schematic diagram of this study. iRGD‐engineered exosomes transport *CPT1A* siRNA specifically to colon cancer cells, which efficiently silence *CPT1A* and serve as a potential carrier to reverse oxaliplatin resistance in drug‐resistant colon cancer.

In conclusion, *CPT1A* silencing showed great potential to suppress FAO and reverse oxaliplatin resistance in colon cancer, as shown in Fig. 8. Blocking *CPT1A* with iRGD‐exo‐si sensitized cells to oxaliplatin with low toxicity and high tumour‐targeting ability, which not only provided an effective approach to treat colon cancer that is oxaliplatin resistant but also propelled the application of siRNA in the clinic. These achievements hold great prospects for clinical applications with low toxicity and high efficiency if iRGD‐exo‐si could be explored and used widely in the future.

## Conflict of interest

The authors declare no conflict of interest. All authors listed have approved of the manuscript and consented for publication.

## Author contributions

DL, HZ and RL performed most of the experiments and wrote the manuscript. TD, TN and MB performed some of the experiments. YY, KZ, JW, JD and SG reviewed and edited the manuscript. YB, GY and BS designed the experiments and edited the manuscript. YB is the guarantor of this work and has full access to all of the data in the study and takes responsibility for the integrity of the data and the accuracy of the data analysis.

### Peer Review

The peer review history for this article is available at https://publons.com/publon/10.1002/1878‐0261.13052.

## Supporting information

Fig. S1. (A–B) The expression of αvβ3 (A) and NRP‐1 (B) in human colon cancer tissues and paired normal tissues was identified by IHC (total *n* = 13, 8 of 13 are were shown in Fig. S1A,B). (C) Validation of oxaliplatin resistance of sw480‐lohp/HCT116‐lohp cell lines by CCK‐8 assay.

## Data Availability

The data sets used and/or analysed during the current study are available from the corresponding author on reasonable request.
