# Corrigendum to: iRGD‐modified exosomes effectively deliver CPT1A siRNA to colon cancer cells, reversing oxaliplatin resistance by regulating fatty acid oxidation

**DOI:** 10.1002/1878-0261.13258

**Published:** 2022-06-08

Lin et al. [1] would like to correct Fig. 4B as errors were introduced in the preparation of these figures for publication. The authors were able to provide to the journal the raw data underlying all experimental replicates. The journal has reviewed replicate raw data and the Editors and Authors confirm that correction of this error has not altered the interpretation of data and did not affect the conclusions presented in the article. The authors sincerely apologise for their mistake and for any inconvenience caused.

**Fig. 4:**
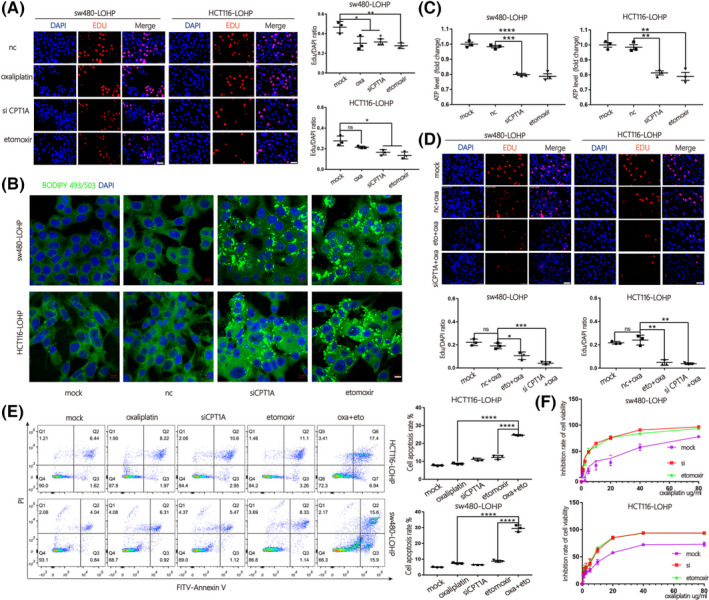
Inhibition of *CPT1A* suppressed FAO and reversed oxaliplatin resistance in colon cancer. (A) An EdU assay was used to evaluate the proliferation of sw480‐lohp/HCT116‐lohp cells after different treatments; red indicates newly proliferating cells (scale bar = 50 μm, *n* = 3, mean ± SEM, *t*‐test). (B) Cell lines with *CPT1A* inhibited by siRNA or etomoxir were pretreated with oleic acid (OA, 200 μm) for 24 h and then cultured in low glucose media RPMI 1640 for an additional 48 h. cells were stained with BODIPY‐493/503 (dyes of fatty acids, 1 μg·mL^−1^, green) and DAPI (blue) and imaged by confocal microscopy (scale bar = 10 μm). (C) Relative ATP production of cells treated with *siCPT1A* or etomoxir was analysed by a CellTiter‐Glo luminescent cell viability assay (*n* = 3, mean ± SEM, *t*‐test). (D) Proliferation analysis of sw480‐lohp/HCT116‐lohp cells treated with a combination of oxaliplatin and *CPT1A* inhibition by EdU assay (scale bar = 50 μm, *n* = 3, mean ± SEM, *t*‐test). (E) Flow cytometry analysis of apoptosis in sw480‐lohp/HCT116‐lohp cells treated with *siCPT1A*, oxaliplatin, etomoxir or etomoxir combined with oxaliplatin (*n* = 3, mean ± SEM, *I*‐test). (F) CCK‐8 test of the inhibition ratio by oxaliplatin in sw480‐lohp/HCT116‐lohp cells treated with *siCPT1A* or etomoxir. *P < 0.05; **P < 0.01; ***P < 0.001; ****P < 0.0001.

The corrected figures are reproduced below. Figure 4B: Corrected to match the correct raw data files. Initially, a different image was shown in panel B for the sw480LOHP mock condition.

## References

[1] Lin D , Zhang H , Liu R , Deng T , Ning T , Bai M , et al. iRGD‐modified exosomes effectively deliver CPT1A siRNA to colon cancer cells, reversing oxaliplatin resistance by regulating fatty acid oxidation. Mol Oncol. 2021;15:3430–46. 10.1002/1878-0261.13052 34213835 PMC8637580

