# Supplementary figures and images for: iRGD‐modified exosomes effectively deliver CPT1A siRNA to colon cancer cells, reversing oxaliplatin resistance by regulating fatty acid oxidation
Source: Mol Oncol. 2021 Jul 22;15(12):3430–46. doi: 10.1002/1878-0261.13052 (PMC8637580; doi:10.1002/1878-0261.13052)

**A**Anti- $\alpha\text{v}\beta 3$ 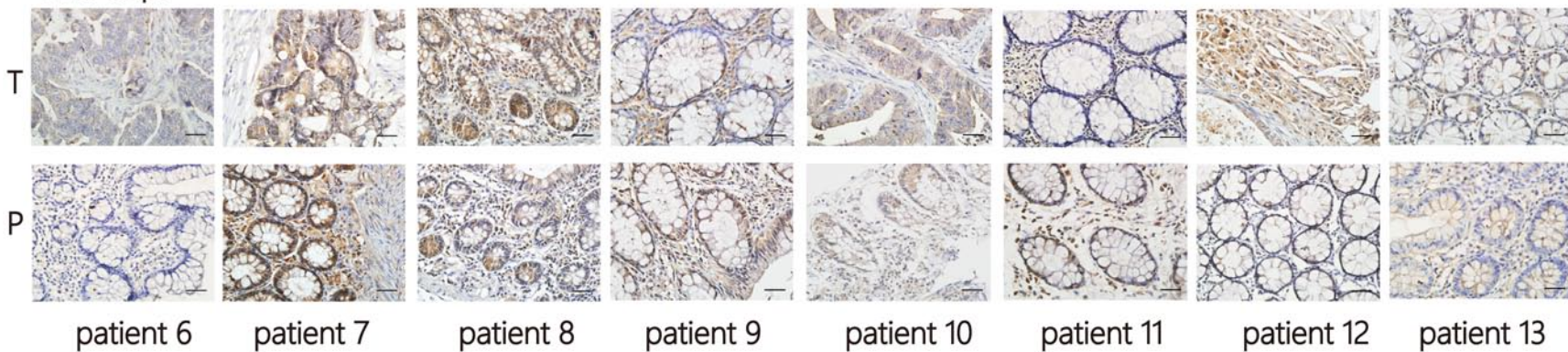**B**

Anti-NRP-1

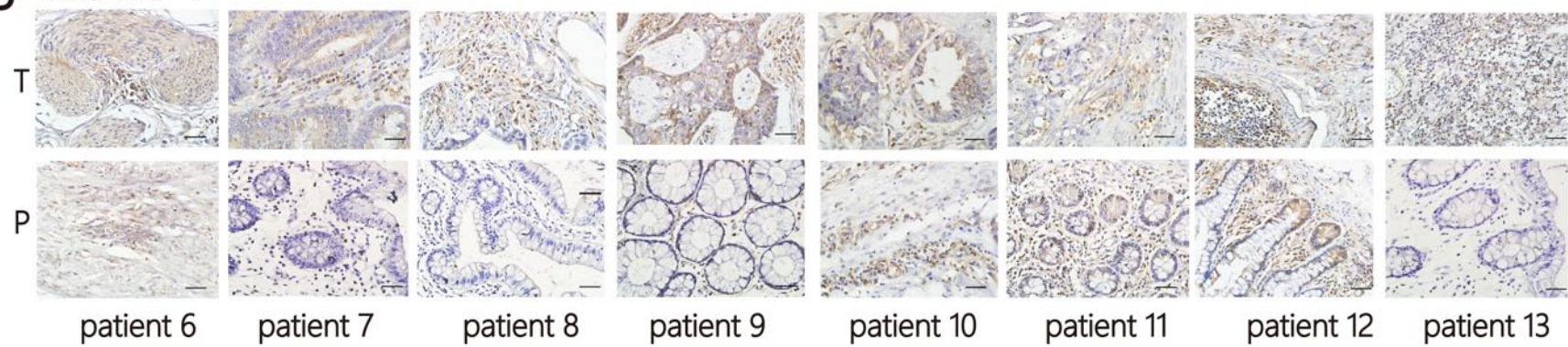**C**

SW480/LOHP

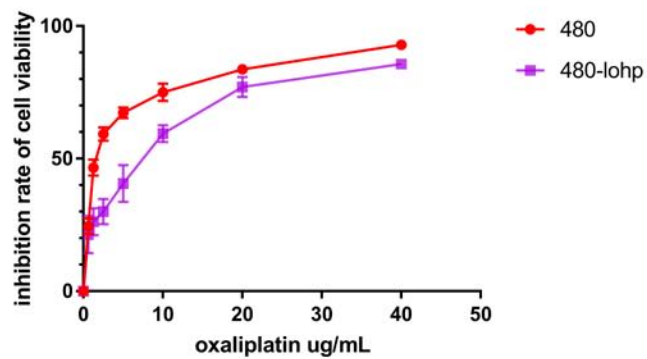

HCT116/LOHP

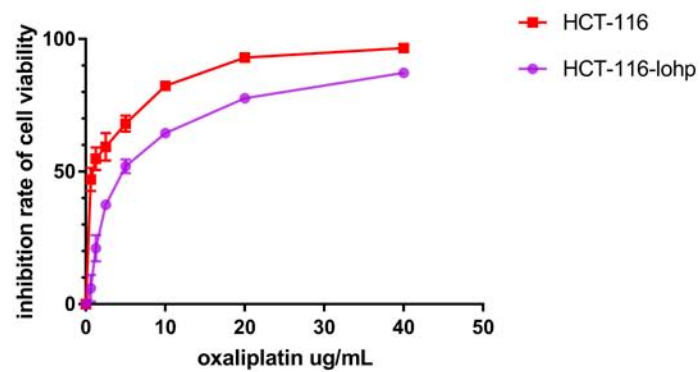

Supplement: Supplementary file 1 — Fig. S1. (A–B) The expression of αvβ3 (A) and NRP‐1 (B) in human colon cancer tissues and paired normal tissues was identified by IHC (total n = 13, 8 of 13 are were shown in Fig. S1A,B). (C) Validation of oxaliplatin resistance of sw480‐lohp/HCT116‐lohp cell lines by CCK‐8 assay. [file MOL2-15-3430-s001.pdf]
